# Thermal Effect and Mechanism Analysis of Flame-Retardant Modified Polymer Electrolyte for Lithium-Ion Battery

**DOI:** 10.3390/polym13111675

**Published:** 2021-05-21

**Authors:** Zhi-Hao Wu, An-Chi Huang, Yan Tang, Ya-Ping Yang, Ye-Cheng Liu, Zhi-Ping Li, Hai-Lin Zhou, Chung-Fu Huang, Zhi-Xiang Xing, Chi-Min Shu, Jun-Cheng Jiang

**Affiliations:** 1 School of Environmental and Safety Engineering, Changzhou University, Changzhou 213164, China; 19083700144@smail.cczu.edu.cn (Z.-H.W.); 19083700288@smail.cczu.edu.cn (Z.-P.L.); 19083700424@smail.cczu.edu.cn (H.-L.Z.); 2 School of Material Science and Engineering, Changzhou University, Changzhou 213164, China; B1900012@smail.cczu.edu.cn (Y.-P.Y.); B20080526@smail.cczu.edu.cn (Y.-C.L.); 3 School of Environmental and Chemical Engineering, Zhaoqing University, Zhaoqing 526061, China; huangchungfu@gmail.com; 4 Department of Safety, Health, and Environmental Engineering, National Yunlin University of Science and Technology, Yunlin 64002, Taiwan; shucm@yuntech.edu.tw

**Keywords:** lithium-ion battery, ethoxy (pentafluoro) cyclotriphosphazene, polymer composite, flame-retardant electrolyte, thermal stability

## Abstract

In recent years, the prosperous electric vehicle industry has contributed to the rapid development of lithium-ion batteries. However, the increase in the energy density of lithium-ion batteries has also created more pressing safety concerns. The emergence of a new flame-retardant material with the additive ethoxy (pentafluoro) cyclotriphosphazene can ameliorate the performance of lithium-ion batteries while ensuring their safety. The present study proposes a new polymer composite flame-retardant electrolyte and adopts differential scanning calorimetry (DSC) and accelerating rate calorimetry to investigate its thermal effect. The study found that the heating rate is positively correlated with the onset temperature, peak temperature, and endset temperature of the endothermic peak. The flame-retardant modified polymer electrolyte for new lithium-ion batteries has better thermal stability than traditional lithium-ion battery electrolytes. Three non-isothermal methods (Kissinger; Kissinger–Akahira–Sunose; and Flynn–Wall–Ozawa) were also used to calculate the kinetic parameters based on the DSC experimental data. The apparent activation energy results of the three non-isothermal methods were averaged as 54.16 kJ/mol. The research results can provide valuable references for the selection and preparation of flame-retardant additives in lithium-ion batteries.

## 1. Introduction

With the rapid development of the electric vehicle industry, more attention has been paid to the endurance of lithium-ion batteries in recent years. Accordingly, high requirements have been established for the energy density of lithium-ion batteries in the electric vehicle market. However, high-energy-density lithium-ion batteries generate substantial heat when releasing energy, which leads to a high risk of thermal runaway. In recent years, accidents involving the spontaneous combustion of electric vehicles have occurred frequently [1,2,3]. On 7 January 2021, a workshop operated by a subsidiary company of Contemporary Amperex Technology Co. exploded due to the thermal runaway of battery waste, resulting in a giant mushroom cloud. Therefore, to enhance the lithium-ion batteries’ essential safety, research on the intrinsic safety of lithium-ion batteries is eagerly demanded. At present, scholars have developed solid-state electrolyte lithium-ion batteries that are safer than liquid-state ones. However, the current solid-state electrolyte lithium-ion batteries face problems, such as short battery cycle life, high solid–solid interface contact resistance, and poor interface compatibility and stability [4]. Nowadays, lithium-ion batteries on the market consist of positive and negative electrodes coated with highly active electrode materials, separators, and electrolytes. To solve the thermal safety problems of lithium-ion batteries, the design and development of electrolyte flame-retardant additives are critical. Adding electrolyte flame-retardant additives is currently one of the most economical and effective methods to reduce the thermal runaway risk of lithium-ion batteries because of their low cost and high performance [5,6,7].

At present, the chief flame-retardant additives include phosphate esters, fluorinated ring triphosphazene, and organic halogenated compounds. In the past few years, phosphate ester flame-retardant additives have shown excellent flame-retardant effects but low electrochemical performance in lithium-ion batteries [8]. Flame-retardant additives derived from organic halogenated compounds are environmentally unfriendly. Furthermore, phosphate esters and organic halogenated compounds need to be added with about 10.0 mass% or more to make the electrolyte non-flammable [9]. Fluorinated ring triphosphazene flame-retardant additives have attracted the attention of researchers because they only need to be added with 5.0 mass% to make the electrolyte non-flammable, and they do not affect electrochemical performance [10,11,12].

Recently, various derivatives of fluorinated ring triphosphazene have been discovered. A new flame-retardant additive, ethoxy (pentafluoro) cyclotriphosphazene (PFPN), has been proposed by researchers. The research into PFPN mainly focuses on the self-extinguishing time (SET) test and the electrochemical performance test of the PFPN as an electrolyte additive of the lithium-ion battery. Lan has indicated that the PFPN additive has sufficient electrochemical compatibility with the graphite anode and improved the cycling performance of the positive electrode of the lithium-ion battery through charge and discharge tests [13]. Liu has pointed out that PFPN can improve the cycle stability of a lithium nickel manganese oxide (LiNi_0.5_Mn_1.5_O_4_) cathode and has a flame-retardant effect [14]. Using SET and critical oxygen index tests, it has become clear that PFPN can reduce electrode polarization and enhance the electrochemical performance of LiCoO_2_ electrodes [15].

However, few researchers have analyzed the thermal hazard and thermal stability of PFPN electrolytes. It is necessary to investigate the thermal properties of lithium-ion battery electrolytes. Hence, in the present paper, differential scanning calorimetry (DSC) and accelerating rate calorimetry (ARC) were used to conduct thermal analyses on standard lithium-ion battery electrolytes and PFPN-added new polymer composite lithium-ion battery electrolytes [16,17,18]. The flame retardancy of PFPN and the thermal stability of PFPN at different temperatures were also analyzed. Based on experimental data, we carried out simulations to determine the thermal decomposition mechanism of PFPN and used different non-isothermal methods to calculate its thermokinetic parameters. According to the experimental results and the thermokinetic models (Kissinger, Kissinger–Akahira–Sunose (KAS), and Flynn–Wall–Ozawa (FWO)), the apparent activation energy (*E*_a_) was then calculated.

## 2. Materials and Methods

### 2.1. Chemicals and Materials

All chemicals and materials were purchased from commercial sources (Battery grade, Aldrich Co.) and used directly without further purification. As an electrolyte salt, lithium hexafluorophosphate (LiPF_6_) has excellent ionic conductivity, solid electrolyte interface (SEI) film formation, and corrosion resistance. At the same time, the nature of the organic solvent in lithium-ion battery electrolytes often determines the nature of the electrolyte. In order to form a high-quality SEI film that is more easily reduced at low potential, ethylene carbonate (EC) with a ring structure and dimethyl carbonate (DMC) with a low viscosity are very good choices. The initial characteristics of common lithium-ion battery electrolyte solvents are shown in Table 1 [19,20]. This experiment used commercial electrolyte LP30 (1.0 M, LiPF_6_ dissolved into a 1:1 (by volume) mixture of EC and DMC) as the standard electrolyte. The literature indicated that the flame-retardant and electrochemical performance of adding 5.0 mass% cyclotriphosphazene flame-retardant additives is the best [13,15]. Therefore, 5.0 mass% PFPN (>98 mass%, TCI) was selected to be added to the standard electrolyte as a flame-retardant electrolyte. Both electrolytes are configured in a glove box (JMS-S1X, Nanjing Jiumen Automation Technology Co., Nanjing, China) with a water and oxygen content of less than 0.2 ppm. An argon atmosphere was used to prevent the electrolyte from reacting with oxygen and water in the air.

### 2.2. Adiabatic Reaction Calorimetry (ARC) Measurement

Studies have shown that PFPN has sound flame-retardant properties, but the thermal behavior and thermal parameters are unclear [13,15]. ARC (ARC254, NETZSCH-Gerätebau GmbH Co., Selb, Germany) experiments were performed on the standard electrolyte and the flame-retardant electrolyte to obtain the thermokinetic parameters and reaction heat of PFPN in the electrolyte under the adiabatic state. The tested electrolyte sample was inserted into a titanium alloy ball, sealed with a thin film, and the operations were carried out in the glove box. The net weight was controlled at 3.0 ± 0.5 g. We selected the heat–wait–search mode for adiabatic testing from the ARC experiments settings. The sample was swiftly heated to 80.0 °C from the room temperature at which the experiment started, and then the temperature was equilibrated for 30.0 min to test the heat release. When the self-heating rate was less than 0.02 °C/min, there was determined to be no exothermal process at that temperature, and then a 5.0 °C increase in temperature was carried out at a rate of 10.0 °C/min to test the exotherm again. If an exothermic reaction was detected, the system adapted the external environment temperature to make it consistent with the reaction system temperature and achieve the pseudo-adiabatic effect. When the temperature reached 350.0 °C, the heat–wait–search mode ended, and the temperature and pressure changes of the reaction system under the adiabatic state were obtained [21].

### 2.3. Differential Scanning Calorimetry (DSC) Measurement

To strengthen the validity of the experimental data measured from ARC, we used the DSC instrument (HP DSC3, Mettler-Toledo Co., Zurich, Switzerland) to examine the standard electrolyte and PFPN-added flame-retardant electrolyte. The obtained specific exothermic characteristics of the new polymer composite flame-retardant electrolyte were further combined with thermokinetic models. The two electrolyte solutions were put into the aluminum DSC sample pan and sealed in a glove box filled with argon gas to prevent external moisture and oxygen from contacting the electrolyte. The net electrolyte content in the sample pan was 6.5 ± 0.5 mg. The samples were heated from 30 to 350 °C at heating rates of 1, 2, 4, 7, and 10 °C/min [22,23,24,25,26]. By comparing the thermal stability parameters (initial reaction temperature (*T*_o_), peak temperature (*T*_p_), and end reaction temperature (*T*_e_), the thermal behavior of the standard electrolyte and flame-retardant electrolyte were evaluated [27,28,29,30].

### 2.4. Kinetic Analysis

Utilizing the thermokinetic models to determine *E*_a_ is a reliable method to reveal electrolyte thermal safety and mechanisms. Based on the thermodynamic data obtained from the DSC experiments, the present paper used the iso-conversional rate method to calculate the thermokinetic parameters of electrolytes. Given that the data were obtained at the same conversion rate (*α*) under different heating rates (*β*) in the same experiment, the *E*_a_ obtained is relatively reliable [31,32]. The present study used three model-free non-isothermal methods (Kissinger, KAS, and FWO) to calculate the *E*_a_ of the essential electrolyte and the flame-retardant electrolyte. The thermal safety evaluation of PFPN in the electrolyte can be better evaluated by comparing the calculation results.

#### 2.4.1. Kissinger Model

The Kissinger method, also known as the maximum method, is applicable for processes that occur under linear heating rate conditions. Given its convenience, it is the most widely used conversion rate method [33,34,35,36]. The method is shown as the following Equation (1):

(1) $$\ln\left(\frac{\beta }{T^{2}}\right)=\ln\left[-\frac{AR}{E_{a}}f^{\prime }\left(\alpha \right)\right]-\frac{E_{a}}{RT}$$

where *A* is the pre-exponential factor; *T* is the reaction temperature, K; *f*(*α*) is the mechanism function, *f*’(*α*) = df(*α*)/d*α*; *R* is the ideal gas constant, *R* = 8.314 J/(mol·K); and *E*_a_ is an exact value only when *f*′(*α*) is a constant. Therefore, *f*′(*α*) needs to be independent of the heating rate.

#### 2.4.2. KAS Method

Using the Coats–Redfern approximation of the temperature integral, the KAS equation was derived. As a conversion rate method, the KAS method improves the accuracy of calculating *E*_a_ [32,33,37]. As shown in the following Equation (2):

(2) $$\ln\left(\frac{\beta }{T^{2}}\right)=\ln\left[\frac{AR}{E_{a}G\left(\alpha \right)}\right]-\frac{E_{a}}{RT}$$

where *G*(*α*) is the reaction function.

#### 2.4.3. FWO Method

This method calculates the same reaction at different heating rates; when *α* is the same value, the *G*(*α*) is specific [33,38,39,40], as shown in the following Equation (3):

(3) $$\ln\beta =\ln\left[\frac{AE_{a}}{RG\left(\alpha \right)}\right]-2.315-0.4567\frac{E_{a}}{RT}$$

When *α* is appointed, the corresponding *T* can be calculated. Therefore, a fixed *α* has corresponding *T* and *β* values. Then *E*_a_ can be calculated according to the above formula. In this method, the error caused by mechanism functions can be avoided because *E*_a_ is obtained directly without utilizing these functions.

## 3. Results and Discussion

### 3.1. Analysis of Results

**Figure 1:**
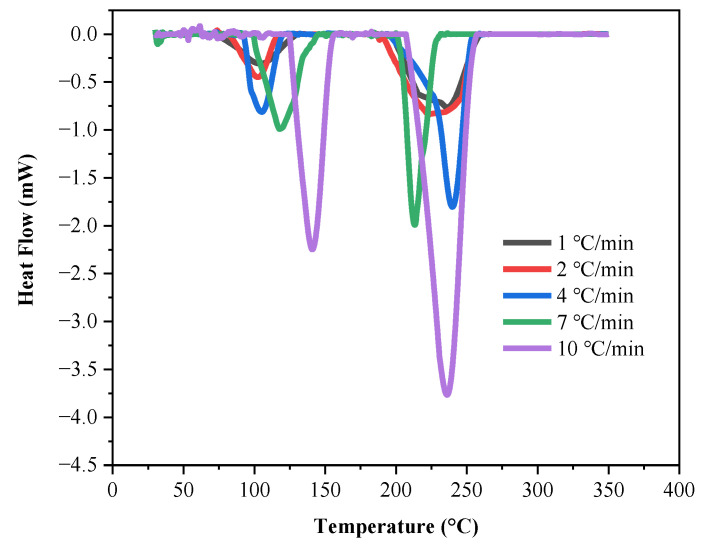
Endothermic curves of LP30 at different heating rates.

**Figure 2:**
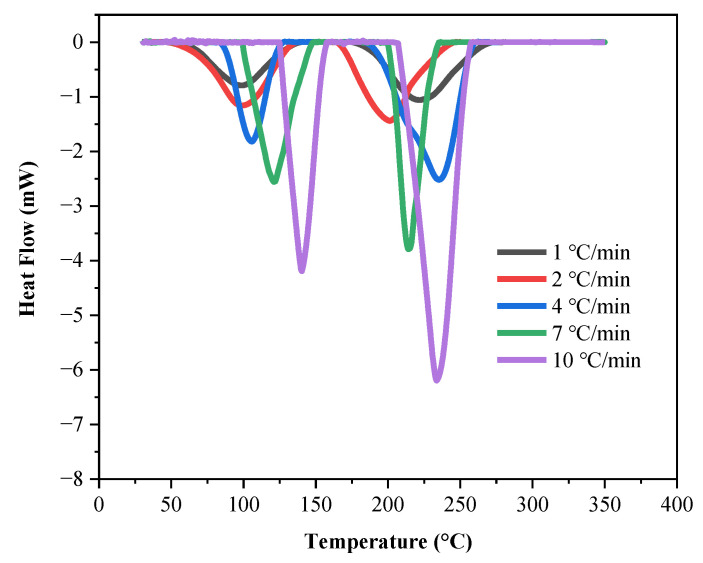
Endothermic curves of LP30 + PFPN at different heating rates.

**Figure 3:**
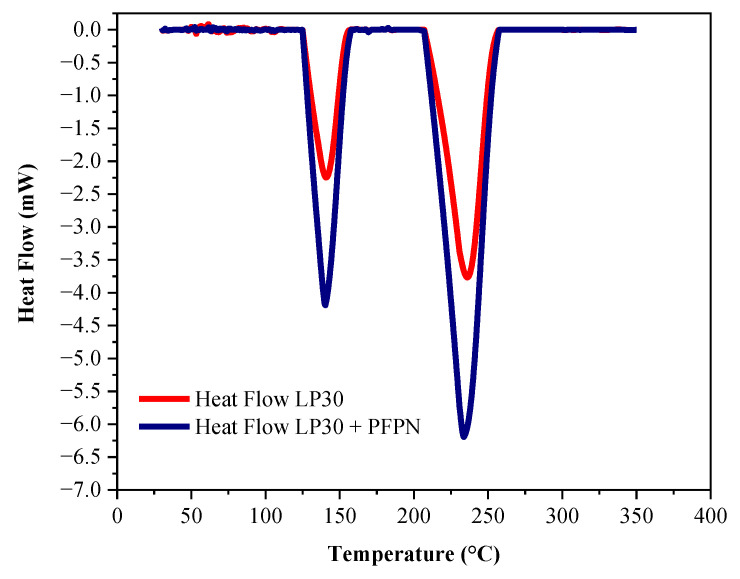
Endothermic curves of LP30 and LP30 + PFPN at *β* of 10 °C/min.

The DSC calorimetry results of LP30 and LP30 + PFPN are shown in Table 2, and Figure 1 and Figure 2. With the increase in *β*, the *T*_o_, *T*_p_, and *T*_e_ in the endothermic peaks of LP30 and LP30 + PFPN increased. With the increase in temperature, two endothermic peaks occurred for LP30 and LP30 + PFPN. The two endothermic peaks of LP30 appeared between 68.67 and 158.33 °C and 192.67 and 258.67 °C. The two endothermic peaks of LP30 + PFPN appeared between 61.33 and 159.33 °C and 178.67 and 260.67 °C. According to the literature, the reason for the first endothermic peak is the decomposition of LiPF_6_, which decomposes into LiF solid and PF_5_ gas in an inert environment [41,42]. The reason for the second endothermic peak is the evaporation of the solvent in the electrolyte. The first peak range is smaller than the second one, indicating that LiPF_6_ requires less energy to decompose, and it is easier to react. As shown in Figure 3, at the same heating rate, the areas of the two endothermic peaks became more significant after adding PFPN, indicating that the reaction heats of the two reactions had become more intense. Besides this, the endothermic peak temperature range widened, implying that the endothermic reaction commenced earlier and the reaction system was safer than the original LP30. It can be inferred from this that PFPN can make the LP30 electrolyte more stable.

**Figure 4:**
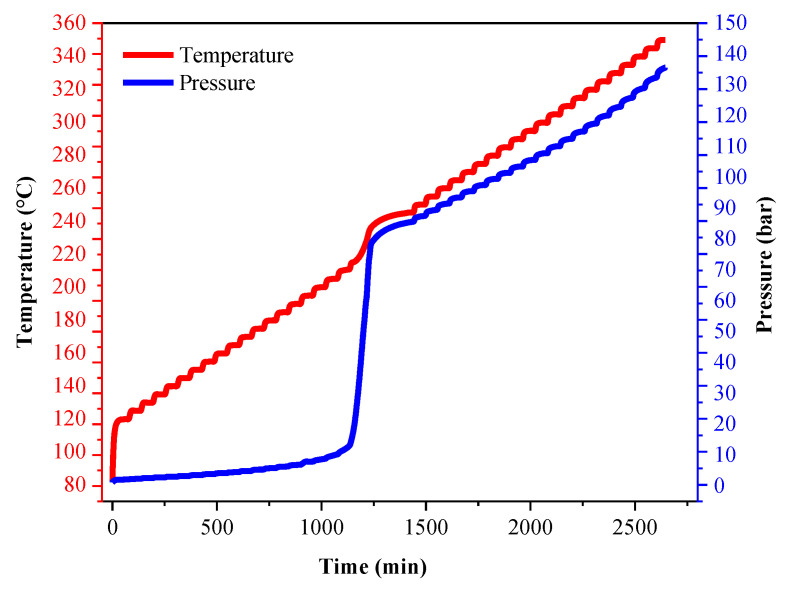
Temperature and pressure versus time (0–2700 min) curves of LP30 + PFPN.

**Figure 5:**
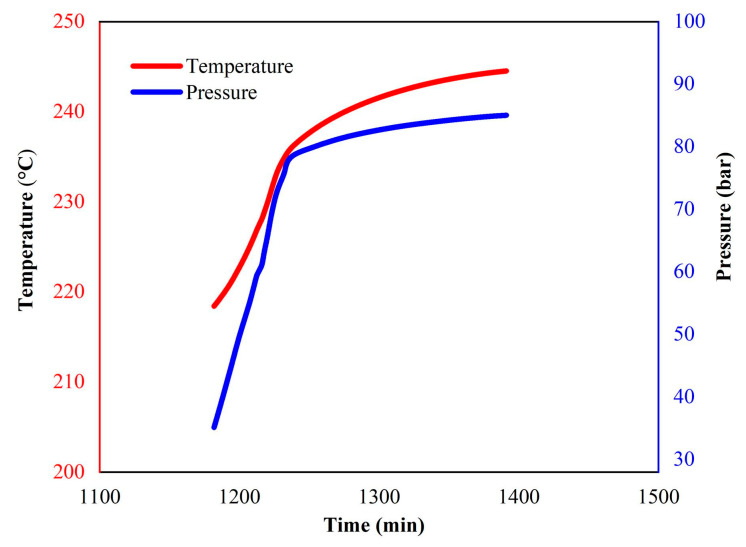
Temperature and pressure versus time (1180–1400 min) curves of LP30 + PFPN.

**Figure 6:**
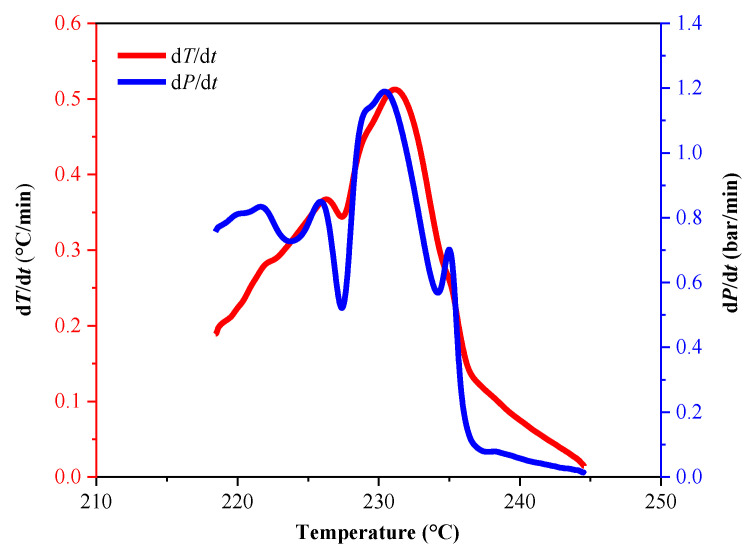
Temperature rise rate and pressure rise rate versus temperature curves of LP30 + PFPN.

**Figure 7:**
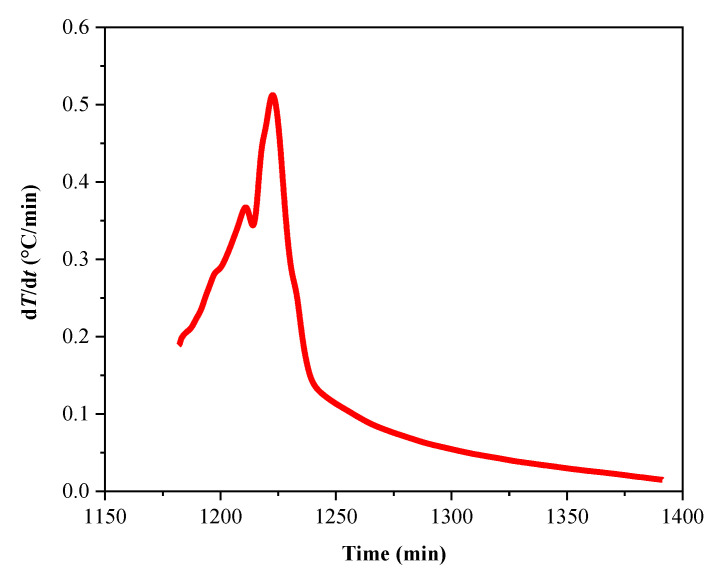
Temperature rise rate time curve of LP30 + PFPN.

To further analyze the thermal decomposition behavior of the LP30 + PFPN electrolyte, we conducted an ARC experiment under pseudo-adiabatic conditions. The characteristic parameters of the experiment are listed in Table 3. As illustrated in Figure 4 and Figure 5, when the experiment had progressed by about 1180 min, the temperature of the reaction system reached about 220 °C. At this time, the temperature of the reaction system began to rise, and the pressure also increased. As shown in Figure 6 and Figure 7, when the experiment had been carried out for about 1225 min, the temperature of the reaction system reached about 231 °C, and the pressure reached approximately 70 bar. At this time, the heating rate and pressure rise rate reached their peak values, which were 0.51 °C/min and 1.2 bar/min, respectively.

The increase in pressure is caused by the vaporization of the solvent in the LP30 + PFPN electrolyte. The temperature at which the pressure in the reaction system started to increase is consistent with the temperature range of the second endothermic peak in the DSC experiment. Because the reaction system is in a pseudo-adiabatic environment, and there is negligible heat loss, it is reasonable to increase the temperature slowly from 220 to 245 °C.

### 3.2. Analysis of Thermodynamic Results

The interior of the lithium-ion battery system is not an adiabatic environment; therefore, this article discussed the effect of PFPN on the thermal stability of LP30. A kinetic model was established, and the kinetic parameters were calculated based on the data of the first endothermic peak in the DSC experiments.

**Figure 8:**
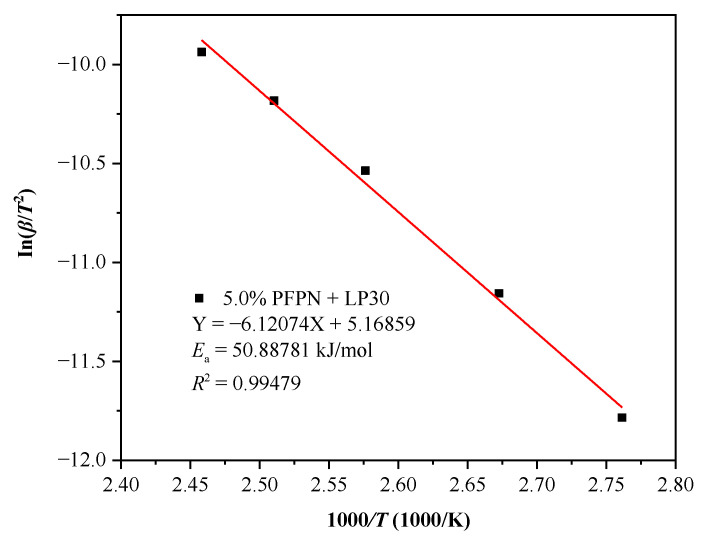
*E*_a_ plots of Kissinger model at different *β* in DSC experiments for LP30 + PFPN.

**Figure 9:**
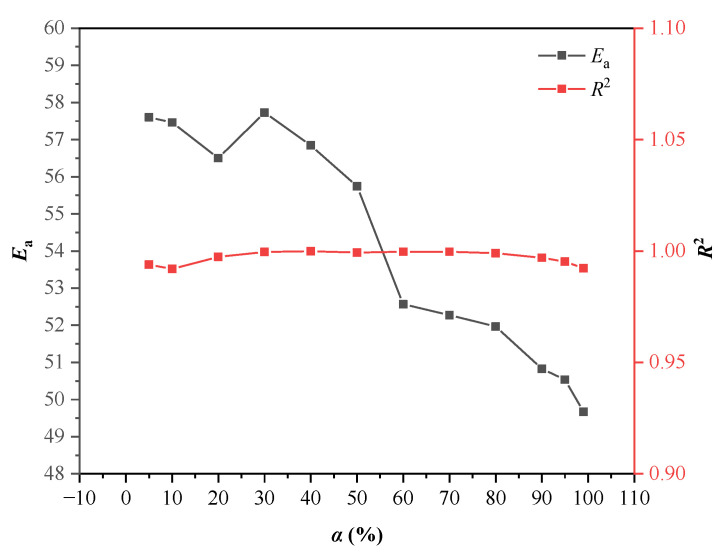
In the DSC experiment, when *β* is equal to 1, 2, 4, 7, and 10 °C/min, the *α*-*E*_a_ and *α*-*R*^2^ polyline of LP30 + PFPN under the KAS model.

**Figure 10:**
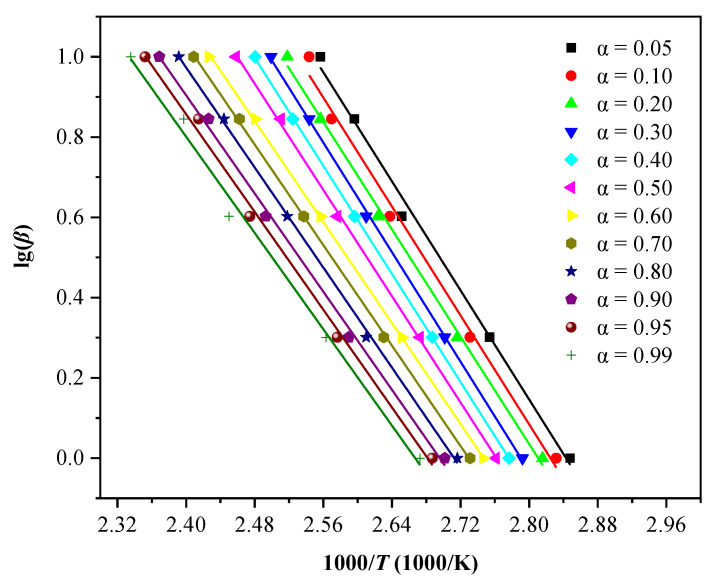
In the DSC experiment, when *β* is equal to 1, 2, 4, 7, and 10 °C/min, differential isoconversional analysis of LP30 + PFPN under the FWO model.

The International Congress on Thermal Analysis and Calorimetry pointed out that the data obtained from multiple heating rates are reliable for thermokinetic establishment. It is also recommended to use the equal conversion rate method to calculate the reaction kinetics [33]. Therefore, we employed the three models of Kissinger, KAS, and FWO to calculate the *E*_a_ through the DSC experimental data at different heating rates (*β* = 1, 2, 4, 7, and 10 °C/min). Thereby, mutual authentications were realized based on the accuracy of the *E*_a_ values. For both KAS and FWO methods, since the choice of baseline is subjective during analysis, we deliberately chose 12 conversion degree values (*α* = 0.05, 0.1, 0.2, 0.3, 0.4, 0.5, 0.6, 0.7, 0.8, 0.9, 0.95, and 0.99) for thermokinetic analysis [43,44]. Figure 8 shows the fitting results based on the Kissinger model using the linear relationship between l n (*β*/*T*^2^) and 1000/*T*. The calculation results show that *E*_a_ was 50.8878 kJ/mol and the determination coefficient *R*^2^ was 0.9948. Figure 9 shows the corresponding *E*_a_ and determination coefficient (*R*^2^) based on the KAS model at different conversion degrees. Then, average values were obtained as ${\bar{E}}_{a}$ = 54.1431 kJ/mol and ${\bar{R}}^{2}$ = 0.9970. Figure 10 demonstrates the fitting results using the linear relationship between l g (*β*) and 1000/*T* at different conversion degrees based on the FWO model. Table 4 showcases the values of *E*_a_ and *R*^2^ calculated based on the fitting results under different conversion degrees of the FWO model. The average values were also obtained as ${\bar{E}}_{a}$ = 57.4436 kJ/mol and ${\bar{R}}^{2}$ = 0.9967. Table 5 displays the *E*_a_ and *R*^2^ calculated by the three models. The *R*^2^ values calculated by the three models were all approaching 1.0, and the *E*_a_ values showed a slight difference, indicating that the *E*_a_ calculated using the three models is relatively reasonable. In conclusion, *E*_a_ can be determined to be 54.1582 kJ/mol by calculating the average of the three values.

## 4. Conclusions

Using the self-synthesized new polymer composite flame-retardant electrolyte, a series of DSC and ARC calorimetry experiments were carried out. Furthermore, a variety of iso-conversion degree models were adopted to calculate the thermodynamic parameters of the new flame-retardant electrolyte. We analyzed the thermal decomposition of LP30 + PFPN and the influence of the flame-retardant additive, PFPN, on the thermal stability of the electrolyte. The experimental results of DSC showed that the values of *T*_o_, *T*_p_, and *T*_e_ are obviously affected by *β* and have a positive relationship with the value of *β*. In an argon atmosphere, LiPF_6_ in the new flame-retardant electrolyte decomposed into LiF solid and PF_5_ gas at ca. 100 °C, and the addition of PFPN can ameliorate the thermal stability of the electrolyte. Three models—Kissinger, KAS, and FWO, were used to calculate the *E*_a_ of LP30 + PFPN, and the calculated results were adjacent, in that *R*^2^ values approached 1. The *E*_a_ results of the three methods were averaged as 54.16 kJ/mol. Therefore, the PFPN-added new polymer composite flame-retardant lithium-ion battery electrolyte has better thermal stability than standard electrolytes.

## Figures and Tables

**Table 1:** The initial characterization of common lithium-ion battery electrolyte solvents.

Organic Solvent	Boiling Point (°C)	Flash Point (°C)	Melting Point (°C)	Viscosity (mPa·s) (25 °C)	Dielectric Constant
Ethylene carbonate (EC)	248	160	37	1.90	89.6
Dimethyl carbonate (DMC)	90	17	2	0.62	2.6
Propylene carbonate (PC)	242	128	−49	2.50	69.0
Diethyl carbonate (DEC)	126	25	−43	0.75	2.8
Ethyl methyl carbonate (EMC)	108	23	−55	0.65	2.9

**Table 2:** Characteristic parameters of LP30 and LP30 + PFPN.

*β* (°C/min)	Mass (mg)	LP30						LP30 + PFPN					
		*T*_o1_ (°C)	*T*_p1_ (°C)	*T*_e1_ (°C)	*T*_o2_ (°C)	*T*_p2_ (°C)	*T*_e2_ (°C)	*T*_o1_ (°C)	*T*_p1_ (°C)	*T*_e1_ (°C)	*T*_o2_ (°C)	*T*_p2_ (°C)	*T*_e2_ (°C)
1	6.5 ± 0.5	68.67	103.33	132.33	192.67	223.00	260.33	61.33	96.67	140.33	178.67	222.00	272.67
2		78.67	103.33	123.33	191.67	223.00	258.00	56.00	97.67	137.00	162.33	200.00	248.67
4		89.00	104.67	122.00	193.33	239.67	262.33	81.33	105.33	129.67	183.00	234.00	264.00
7		95.45	117.85	146.55	195.90	212.70	234.47	95.45	121.00	150.058	196.25	214.10	236.85
10		121.67	140.67	158.33	202.33	234.67	258.67	122.00	139.00	159.33	204.33	233.67	260.67

*T*_1_ represents the first endothermic peak; *T*_2_ represents the second endothermic peak.

**Table 3:** ARC test parameters for LP30 + PFPN.

m_s_ (g)	m_ball_ (g)	Material	Temperature (°C)	Heating Rate (°C/min)	Exothern Threshold (°C/min)	Waiting Time (min)	Temperature Increment (°C)
3.346	21.000	Hastelloy	80–350	10	0.02	30	5

**Table 4:** *E*_a_ and *R*^2^ under different *α* based on the FWO model.

*α*	*E*_a_ (kJ/mol)	*R* ^2^
0.05	60.5039	0.9931
0.1	60.4064	0.9909
0.2	59.5369	0.9969
0.3	60.7497	0.9995
0.4	59.9467	0.9999
0.5	58.9380	0.9992
0.6	55.9757	0.9997
0.7	55.7361	0.9996
0.8	55.4792	0.9988
0.9	54.4398	0.9966
0.95	54.1983	0.9948
0.99	53.4124	0.9917

**Table 5:** ${\bar{E}}_{a}$ and *R*^2^ values obtained by Kissinger, KAS and FWO methods.

	${\bar{E}}_{a}$ (kJ/mol)	${\bar{R}}^{2}$
Kissinger	50.8878	0.9948
KAS	54.1431	0.9970
FWO	57.4436	0.9967

## Data Availability

The data presented in this study are available on request from the corresponding author.
